# Humidity-Controlled Growth of Self-Healing Poly(ethylenimine)/Poly(acrylic Acid) (PEI/PAA) Multilayer Films

**DOI:** 10.1021/acsapm.6c00365

**Published:** 2026-05-30

**Authors:** Shirley M. V. da R. Hossack, Jonathas P. Siqueira, Giovanni Budroni Netto, Antonio Riul, Varlei Rodrigues

**Affiliations:** 28132 Institute of Physics Gleb Wataghin - UNICAMP, Campinas, São Paulo 13083-510 Brazil

**Keywords:** layer-by-layer assembly, self-healing, polyelectrolyte films, water content, humidity-controlled growth

## Abstract

Functional polymer coatings with tunable electrical and surface properties are central to applications in organic electronics, sensing, and practical coatings, where durability and self-healing (SH) capability are also critical for reliable operation. A good candidate for these applications is Poly­(ethylenimine)/poly­(acrylic acid) (PEI/PAA) layer-by-layer (LbL) films with intrinsic SH ability, whose properties are strongly influenced by the amount of water retained within the multilayer structure during growth. In this paper, we investigate PEI/PAA multilayers deposited under controlled relative humidity (RH = 50%, 70%, 90%) to clarify the role of water in film formation and performance. Films deposited at 50% and 70% RH exhibit capacitances of approximately 1.06 nF and 0.70 nF, respectively, while those grown at 90% RH reach approximately 5 nF. Electrical transport shows a pronounced negative differential resistance (NDR) peak at around 1.3 V, strongest in the 90% RH film. Through terahertz time-domain spectroscopy (THz-TDS), we independently confirm hydration differences through changes in the dielectric constant and, consequently, the refractive index, which manifest as relative delays of the transmitted ultrashort terahertz pulses. Surface characterization reveals a increase in roughness, from 1.8 ± 0.2 μm to 14.8 ± 3.1 μm, accompanied by enhanced hydrophobicity (contact angle rising from 68° to 90°). Only the 90% RH film exhibits complete self-healing within 10 min. Overall, humidity-controlled deposition provides a simple, scalable strategy to tune dielectric response, surface functionality, and SH performance in weak polyelectrolyte multilayers. Such control enables the design of reproducible, multifunctional coatings for next-generation soft-electronic and sensing applications.

## Graphical abstract

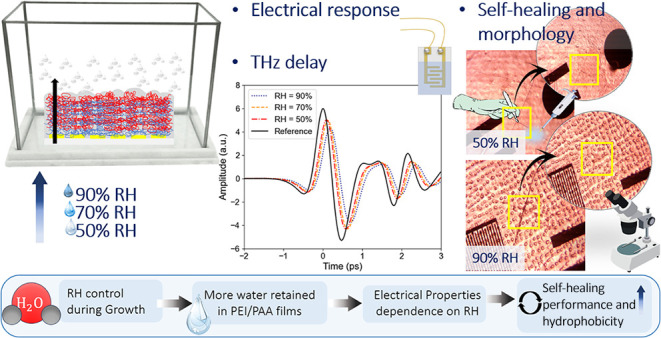

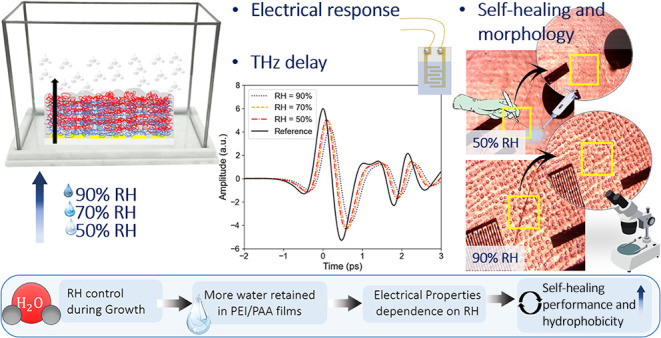

## 1 Introduction

Soft electronics represent a rapidly expanding research field due to their potential in flexible, stretchable, and often biocompatible devices. Applications include health monitoring,^1^ environmental sensing,^2^ agriculture^3,4^ food-quality monitoring (e.g., electronic tongues)^5,6^ as well as electronic skin and wearable platforms.^1,7,8^

These technologies require multifunctional materials with finely tuned electrical, mechanical, and optical properties.^9^ Among the desirable attributes, self-healing (SH) capability is critical, as it improves device robustness and prolongs operational lifetime by repairing mechanical damage.^10–16^ SH materials are generally classified as extrinsic or intrinsic.^17^ Extrinsic systems incorporate microcapsules or vascular networks that release healing agents upon damage, typically enabling only a limited number of repair cycles. Intrinsic SH materials, in contrast, rely on reversible interactions within the polymer matrix and can be activated by external stimuli such as heat or moisture to restore structural integrity.^18^ Materials containing reversible bonds have been studied for decades and remain essential due to their ability to autonomously recover mechanical function under appropriate conditions.^19^

Poly­(ethylenimine)/poly­(acrylic acid) (PEI/PAA) multilayer films have been extensively explored and well-characterized due to the intrinsic SH property^20^ that arises from reversible ionic interactions and hydrogen bonding between weak polyelectrolytes.^21^ Upon water uptake, polymer chains become more mobile, enabling the repair of mechanical damage. Films assembled by Layer-by-Layer (LbL) deposition of weak polyelectrolytes such as PEI and PAA typically show exponential growth in the early stages, followed by a transition to a quasi-linear regime. These growth modes and the corresponding SH behavior depend strongly on solution pH, which governs charge density, ionic strength, and the degree of chain interpenetration.^22,23^ In the weakly interacting regime, enhanced chain mobility enables both SH and the *in-and-out* process, in which solution chains partially diffuse into and redistribute within the growing film before steady-state deposition is reached.^20^

Over the past decade, PEI/PAA-based systems have been extensively explored for a wide range of applications due to their versatility and strong responsiveness to environmental conditions. They have been used in drug-delivery platforms^24^ and as bioadhesive powders that, upon contact with water or humidity, promote healing in medical contexts such as wound and ulcer treatment,^25^ gastrointestinal repair,^25^ arterial bleeding control,^26^ and bone regeneration.^27,28^ These SH adhesives perform effectively on wet surfaces^29^ and exhibit tunable electrical^30^ and mechanical^31^ properties. In addition, PEI/PAA responds to pH variations in the surrounding environment^25^ and has been employed to enhance membrane performance.^10,32^ Depending on the targeted functionality, it can be used either alone or combined with other components to further tailor its properties. Self-healing LbL systems based on PEI/PAA have also been successfully integrated into functional sensing platforms, such as electronic tongues capable of distinguishing chemical species while maintaining electrical performance after healing cycles. These examples highlight the potential of combining electrical functionality with intrinsic self-healing in soft electronic systems.^33^

Film growth regimes are influenced by several parameters, including solution pH, molecular weight, and salt concentration, each affecting chain stretching, adsorption density, and interpenetration. For weak polyelectrolytes, these factors have an even greater impact on the final film properties since changes in pH alter the degree of protonation and, consequently, the electrostatic interactions driving adsorption and layer formation. Additionally, because both PEI and PAA are hygroscopic^34,35^ water uptake during deposition or postprocessing significantly impacts electrical and mechanical performance. Absorbed water enhances chain mobility and screens electrostatic interactions, influencing SH, ion transport, and dielectric response.^36^

Previous studies have demonstrated that relative humidity plays an important role in determining the structure and properties of polyelectrolyte multilayers. For example, humidity-dependent swelling and structural rearrangements have been widely reported, including in strong polyelectrolyte systems, where water uptake and film thickness depend on relative humidity (RH).^37^ In weak polyelectrolyte multilayers, the role of humidity is even more pronounced due to the sensitivity of ionization and intermolecular interactions to hydration. Several works have also highlighted the importance of humidity history and preconditioning. Secrist and Nolte showed that poly­(allylamine hydrochloride) and poly­(acrylic acid) multilayers exhibit swelling/deswelling hysteresis and retain a memory of prior humidity exposure, indicating that the internal structure depends on RH history.^38^ Similarly, Tanchak and Barrett demonstrated that ambient humidity prior to swelling strongly affects transport dynamics and equilibration times in weak polyelectrolyte multilayers.^39^ These findings indicate that hydration conditions can influence both structure and dynamics of polyelectrolyte multilayers. However, in these studies, relative humidity is treated as an external or postdeposition variable, rather than as a controlled parameter during film growth. A systematic investigation of how relative humidity during layer-by-layer assembly influences the resulting structural, electrical, and self-healing properties of weak polyelectrolyte multilayers remains limited. In this work, we address this gap by investigating PEI/PAA multilayers grown under controlled relative humidity conditions, and by correlating RH during deposition with electrical response, morphology, and self-healing behavior.

In this context, the present work aims to understand how relative humidity during LbL assembly affects the structural and electrical properties of weak polyelectrolyte multilayers. Such understanding is essential for the rational design of materials for applications in soft electronics and sensing systems, where hydration plays a critical role.

## 2 Experimental Section

### 2.1 Materials

Poly­(acrylic acid) (PAA, M_V_ = 1,250,000, CAS No. 9003-01-4) and poly­(ethylenimine) (PEI, analytical standard, 50% (w/v) in water, CAS No. 900298-6) were purchased from Sigma-Aldrich (St. Louis, MO, USA). Stock solutions for film assembly were prepared at a concentration of 4 mg/mL. The pH of the PEI solution was adjusted to 9.1 using 1 M HCl, while the PAA solution was used as prepared, with a measured pH of 3.5. Ultrapure water (Arium Comfort Sartorius System, 18.2 MΩ·cm) was used for all preparations. For substrate hydrophilization, ultrapure water, H_2_O_2_, and NH_4_OH were used. Sodium chloride (NaCl), Lithium chloride (LiCl), and potassium sulfate (K_2_SO_4_) were employed to control relative humidity (RH). Substrates consisted of gold interdigitated electrodes (IDEs) patterned on borosilicate glass, each containing 30 pairs of digits, 3 mm in length, 40 μm in width, and spaced 40 μm apart.

### 2.2 Layer-by-Layer Assembly

Prior to deposition, substrates were hydrophilized to ensure a clean, negatively charged surface. They were immersed for 10 min at 90 °C in a 5:1:1 mixture of ultrapure water, ammonium hydroxide (NH_4_OH), and hydrogen peroxide (H_2_O_2_).^40^ Film assembly was carried out using the LbL technique, based on the alternating adsorption of oppositely charged polyelectrolytes.^41,42^ Each deposition cycle consisted of immersion in the cationic solution (PEI), rinsing with water, immersion in the anionic solution (PAA), and a final rinsing step. Rinsing was essential for removing weakly adsorbed species.^43^ The entire deposition process was automated using a custom-built system that controlled all parameters. Substrates were immersed and withdrawn at controlled speeds of 10 mm·s^–1^ and 60 mm·s^–1^, respectively. Immersion times were 15 min in each polyelectrolyte solution, followed by a 15 min drying period. A schematic of the automated system is shown in Figure 1.

**Figure 1:**
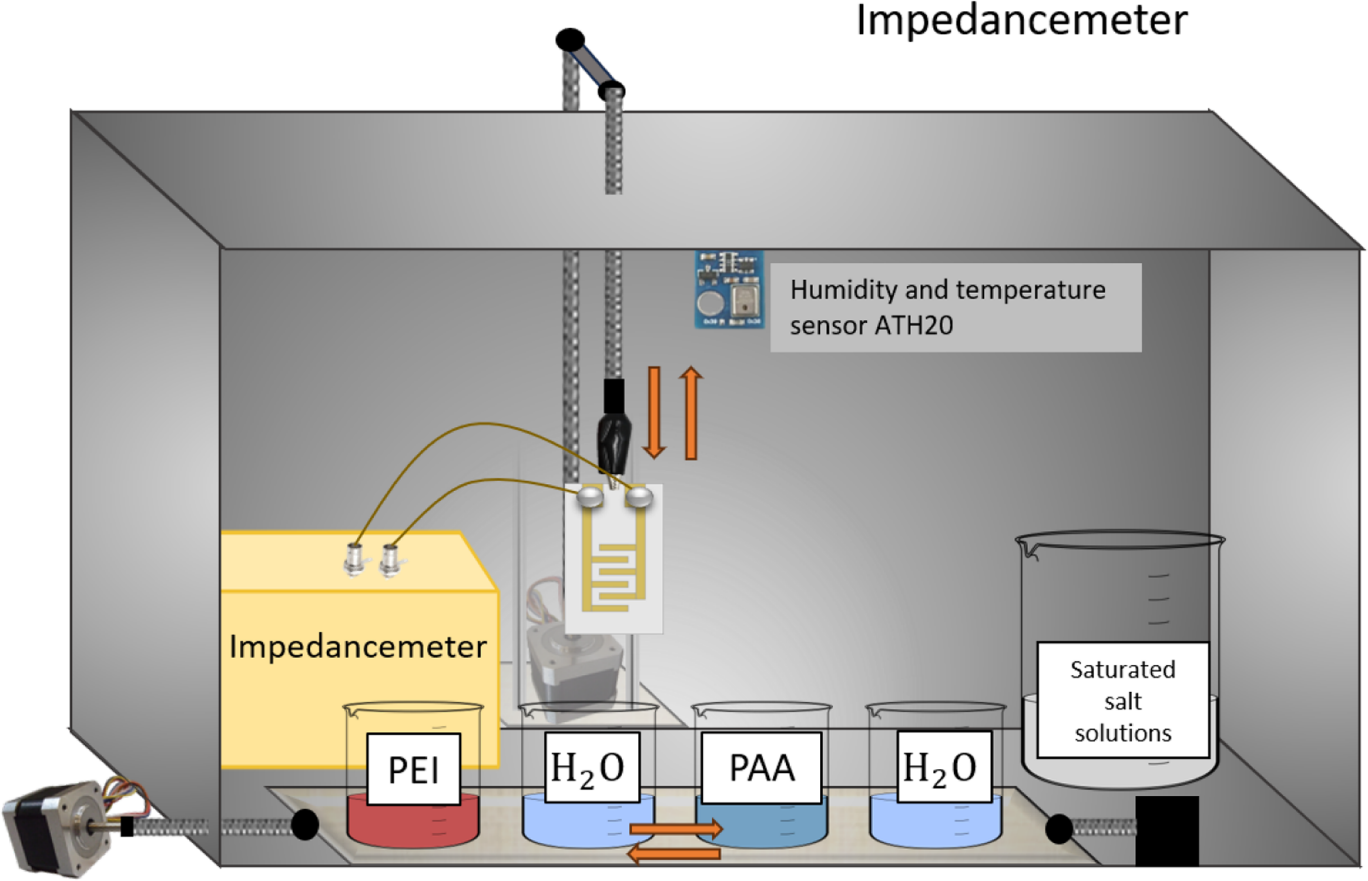
Schematic of the automated Layer-by-Layer deposition system. The deposition chamber is an aluminum enclosure sealed with plastic film to maintain constant relative humidity. Stepper motors and the Arduino controller were located outside the enclosure to prevent electrical interference with *in situ* impedance measurements.

### 2.3 Relative Humidity Control and *In Situ* Monitoring

During the LbL film growth, impedance measurements were carried out after each drying step. Temperature and relative humidity (RH) were continuously monitored using an AHT20 sensor connected to an Arduino controller.

Humidity conditions were adjusted by placing saturated salt solutions inside a sealed aluminum enclosure, together with polyelectrolyte and rinsing solutions. While saturated salts are commonly associated with well-defined equilibrium RH values^44,45^ these values apply to closed systems containing only the salt solution. In the present configuration, the enclosure also contains additional liquid phases, which modify the vapor equilibrium and prevent direct correspondence with standard salt–RH values.

Therefore, in this work, RH conditions are defined exclusively based on direct sensor measurements rather than nominal values associated with the salts. Prior to each experiment, the chamber was allowed to reach a stable RH (variation <1% over 1 h), typically requiring 2–6 h. All experiments were performed at 20 °C. The measured RH values during deposition were (50 ± 2)%, (70 ± 2)%, and (90 ± 2)%, obtained using LiCl (with silica gel), NaCl, and K_2_SO_4_, respectively, to adjust the humidity range. The reported uncertainties correspond to the standard deviation of RH measurements recorded during each deposition run and the variation in RH during deposition remained within the measurement uncertainty of the sensor.

### 2.4 Characterization

#### 2.4.1 Electrical Characterization

*In-situ* impedance measurements were performed using a custom-built impedancemeter previously described in ref 46. The excitation frequency was 1 kHz, and data acquisition was carried out using a Lock-in amplifier (SR830, Stanford Research Systems, Sunnyvale, CA, USA). Electrical transport measurements were performed after deposition at room temperature and ambient RH. Current–voltage (I–V) curves were obtained using a 6484 picoammeter (Keithley Instruments, Cleveland, OH, USA), sweeping the voltage from −3 to +3 V at 5 mV/s, consistent with previous studies.^36^

#### 2.4.2 Optical Characterization: THz-TDS

Terahertz time-domain spectroscopy (THz-TDS) was performed in transmission mode using quartz substrates, both as a reference and support for the PEI/PAA films. In the THz-TDS technique, the temporal profile of a single-cycle terahertz pulse transmitted through a material is precisely measured, allowing minute changes in the index of refraction and dielectric constant to be detected through relative temporal delays when comparing THz pulses transmitted through different film samples. Due to the considerably lower oscillation frequency of terahertz radiation compared to optical frequencies, refractive index variations measured in the THz range are significantly more representative of changes in the DC dielectric constant than those observed at optical frequencies. For accurate characterization of the temporal profile of the THz pulses, a short temporal window ranging from −2 to 3 ps was used, with temporal steps of 20 fs, in order to obtain high sensitivity in resolving relative temporal delays between pulses transmitted through films grown under different RH levels. The central frequency (ω_0_) of the THz pulse spectrum was obtained from a Gaussian fit of the reference signal, resulting in a value of approximately 0.9 THz. The delay of the transmitted pulse relative to the reference was analyzed as a signature of hydration-induced enhancement of the dielectric response.

The complex dielectric permittivity (*ε*(ω)) is related to the refractive index (n) and to the extinction coefficient (κ) by

1 $$\mathrm{\varepsilon ̃}\left(\omega \right)={[n(\omega )+i\kappa (\omega )]}^{2}$$

For a very thin film of thickness *d* that is weakly dispersive in the THz range, the delay at the amplitude maximum of a broadband single-cycle pulse can be approximated by

2 $$\Delta t=\frac{d}{c}\left[n_{\mathrm{film}}\left(\omega_{0}\right)-n_{\mathrm{ref}}\left(\omega_{0}\right)\right]$$

where c is the speed of light in a vacuum, and *n*_film_(ω_0_) and *n*_ref_(ω_0_) are the refractive index of the sample and the reference, respectively, at the central frequency of the THz pulse spectrum. As demonstrated by Ludlam et al.,^47^ the THz-range dielectric constant of thin polymer films such as Nafion is highly sensitive to hydration state, supporting the analysis presented here. Comparable THz data for LbL PEI/PAA films are not yet available.

It should be noted that THz measurements were performed on films deposited on quartz substrates, whereas electrical and morphological characterizations were carried out on films grown on gold interdigitated electrodes on borosilicate glass. While substrate surface chemistry can influence the initial stages of LbL growth, the films investigated here are in the exponential growth regime and reach comparable thicknesses (≈20–22 μm), where bulk properties are expected to dominate. Therefore, the THz results are interpreted as providing complementary information on hydration-related effects, although direct quantitative comparisons between techniques should be made with caution.

#### 2.4.3 Surface Characterization

Film thickness and surface roughness were measured using a profilometer (Dektak 150, Veeco Instruments, Plainview, NY, USA). Thickness was obtained by creating and scanning a step on each film. For each sample, five line scans of 1 mm length were acquired (120 s per scan, 0.028 μm/sample, 3 mg stylus force), and the reported thickness and roughness correspond to the averages of the five scans. SH was evaluated by cutting the films with a surgical scalpel, immersing the damaged samples in water for 10 min, and imaging the recovery using a digital microscope (AM3113T, Dino-Lite, Torrance, CA, USA). Contact angle measurements were performed using a goniometer (DSA 100, Kruss).

## 3 Results and Discussion

The growth and properties of PEI/PAA LbL films were investigated as a function of relative humidity during deposition. PEI/PAA is highly sensitive to water, readily absorbing moisture during assembly; thus, variations in drying conditions alter the amount of water retained in the multilayers. To probe these effects, *in situ* impedance measurements monitored the growth process, while current–voltage (I–V) curves provided insight into charge transport. Time-domain terahertz spectroscopy (THz-TDS) served as an independent probe of hydration, and complementary morphological and SH analyzes allowed us to correlate water content with structure and function.

### 3.1 Capacitance during Film Growth

Figure 2 shows the capacitance as a function of the number of deposited layers (*n*) for films grown at 50%, 70%, and 90% RH. Two distinct regimes are evident. During the first ∼15 layers, the capacitance exhibits irregular oscillations characteristic of the exponential growth regime and the “in-and-out” diffusion process typical of weak polyelectrolytes.^23^ Beyond this regime, the films enter a linear growth phase in which capacitance increases steadily with *n*.

**Figure 2:**
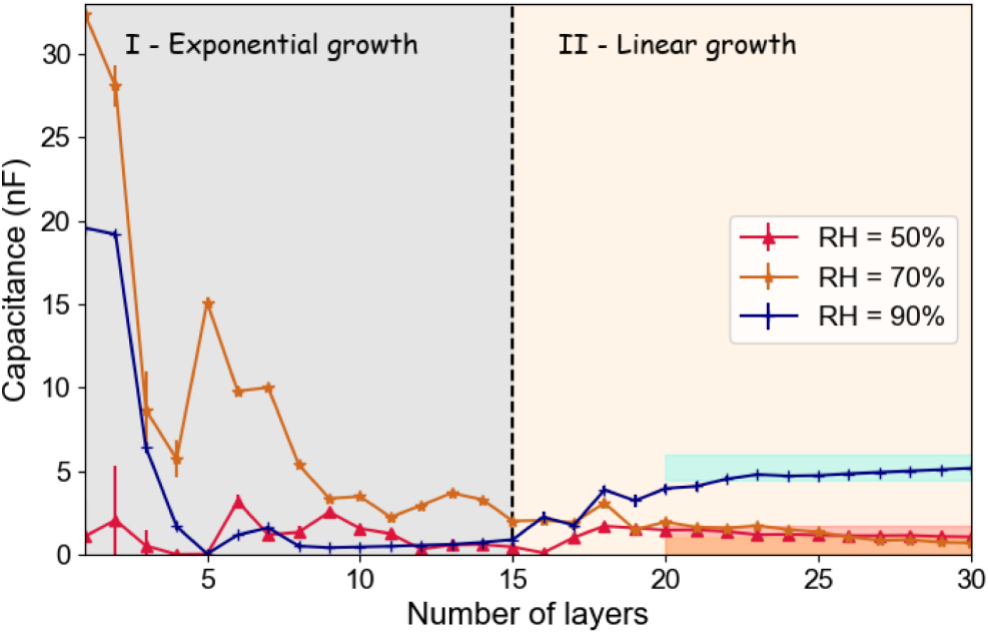
Capacitance (*C*) as a function of the number of PEI/PAA layers (*n*) for films grown at different RH. Red triangles: 50%; orange stars: 70%; blue line: 90%. Two regimes are observed: exponential growth in the first ∼15 layers and linear growth thereafter. Error bars represent the statistical uncertainty from 40 measurements acquired after each drying step.

After the initial 15 layers, corresponding to the exponential growth regime, the films transition to a quasi-steady regime. In this region, the capacitance does not increase monotonically with the number of layers. For films grown at 90% RH, the capacitance increases up to approximately 23 layers and then approaches a plateau. In contrast, films grown at 50% and 70% RH exhibit lower capacitance values with weaker trends, including slight decreases at higher layer numbers. Films grown at 50% and 70% RH stabilize at (1.06 ± 0.01) nF and (0.70 ± 0.04) nF, respectively, whereas those grown at 90% RH reach (5.17 ± 0.05) nF, nearly five times higher. Although a monotonic increase in capacitance with RH might be expected from a simple dielectric model based only on water content, the observed ordering between the 50% and 70% RH films (with the 50% RH film showing higher capacitance than the 70% RH film) indicates that additional factors must be involved.

In weak polyelectrolyte multilayers, hydration can influence not only the amount of absorbed water but also ionic cross-linking, chain interpenetration, porosity, and the balance between bound and mobile species. Moreover, previous studies on PAH/PAA multilayers have shown that hydration can induce structural rearrangements and hysteresis, leading to states that depend on both humidity exposure and relaxation kinetics.^38^ In such systems, electrostatic cross-linking can hinder structural relaxation, resulting in nonequilibrium configurations and history-dependent properties.

Therefore, the higher capacitance observed for films grown at 90% RH is consistent with increased hydration, but the nonmonotonic behavior between 50% and 70% RH suggests that the effective dielectric response is governed by coupled hydration–structure effects rather than by water content alone.

### 3.2 Electrical Transport

Figure 3a shows I–V curves for films grown under different RH conditions. All samples exhibit hysteresis, typical of polymeric films containing trapped charges. Additionally, the curves display regions of negative differential resistance (NDR), in which the current decreases as the applied voltage increases.^48^ This behavior occurs in a voltage range close to the thermodynamic threshold of water electrochemical processes^49^ and is more pronounced in the more hydrated films, indicating that water plays an important role in the observed transport response, consistent with previous observations in PEI/PAA films.^36^ The underlying microscopic mechanism is not directly resolved by the present measurements. Charge trapping may contribute to the low-voltage response in these hydrated films. As the applied voltage increases, the transport response deviates from the initial trend and an NDR feature emerges near 1.3 V, but the present data do not allow us to determine whether this reflects electrochemical processes, space-charge effects, ionic polarization, or a combination of these contributions. Accordingly, the NDR behavior is interpreted here more generally as a manifestation of hydration-dependent charge transport, potentially involving coupled effects of charge trapping, space-charge formation, ionic polarization, and/or electrochemical processes.

**Figure 3:**
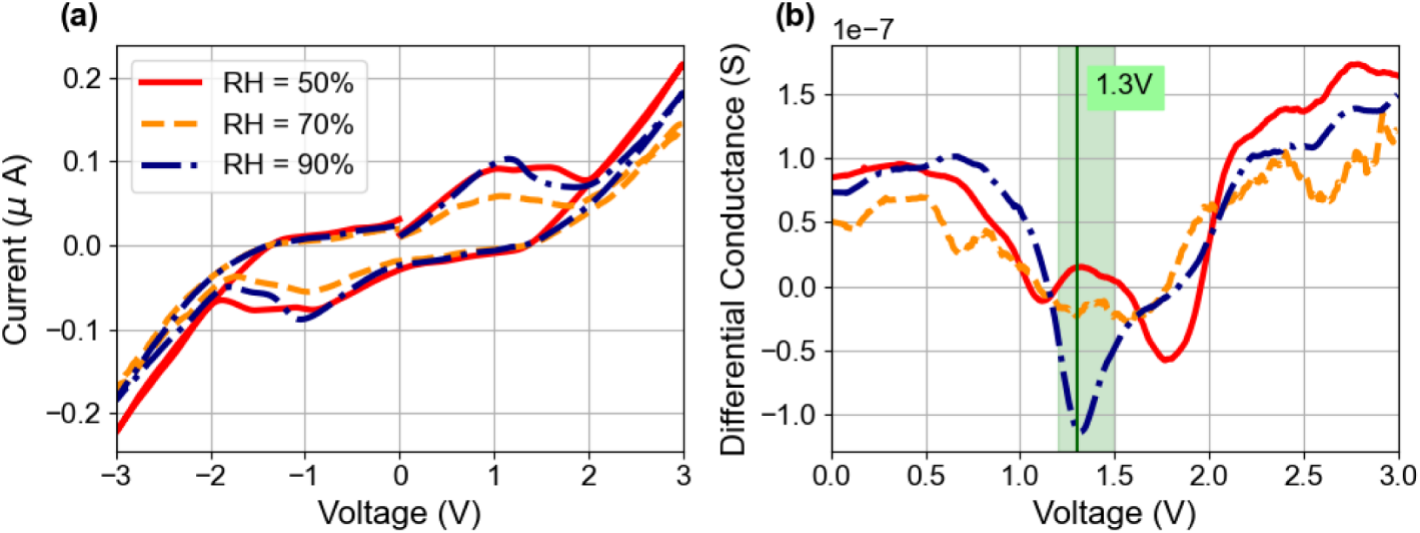
(a) I–V curves of PEI/PAA films grown at 50%, 70%, and 90% RH, showing hysteresis and NDR. (b) Differential conductance (*G* = d*I*/d*V*) for 50% (red) and 90% (blue) RH films, highlighting the pronounced NDR peak around 1.3 V.

Differential conductance (*G* = d*I*/d*V*) curves (Figure 3b) highlight the NDR peak at ∼1.3 V. The peak is strongest for the 90% RH film, while the 50% RH sample exhibits a weaker peak shifted to higher voltages. This shift is consistent with a reduced contribution of hydration-dependent transport processes in drier films, although the present data do not allow a unique microscopic assignment. The 70% RH film shows a distinct intermediate behavior, with the weakest NDR amplitude but a peak position closer to that of the 90% RH sample, indicating that the evolution of the NDR response with RH is not strictly monotonic. This nonmonotonic behavior suggests that the contribution of hydration to charge transport does not scale linearly with RH. Instead, intermediate humidity conditions appear to correspond to a distinct regime in which the balance between ionic mobility, electrostatic screening, and structural organization differs from both lower and higher RH conditions. This interpretation is consistent with the capacitance results, which also exhibit nonmonotonic behavior, indicating that the electrical response is governed by coupled hydration–structure effects rather than by water content alone.

### 3.3 THz Spectroscopy

THz-TDS measurements provided an independent assessment of hydration in the PEI/PAA multilayers through the characterization of the low-frequency dielectric response enhancement with increasing RH under which the films were grown. Figure 4 shows the transmitted THz pulses for the films deposited on quartz, together with the quartz reference. The temporal position of each pulse maximum was extracted by Gaussian fitting, yielding delays of (91.0 ± 8.5) fs, (127.1 ± 8.5) fs, and (183.4 ± 8.5) fs for films grown at 50%, 70%, and 90% RH, respectively. The uncertainty (±8.5 fs) is dominated by the translation stage positioning uncertainty (0.9 μm → 6 fs per measurement), combined as the root sum square for the delay difference.

**Figure 4:**
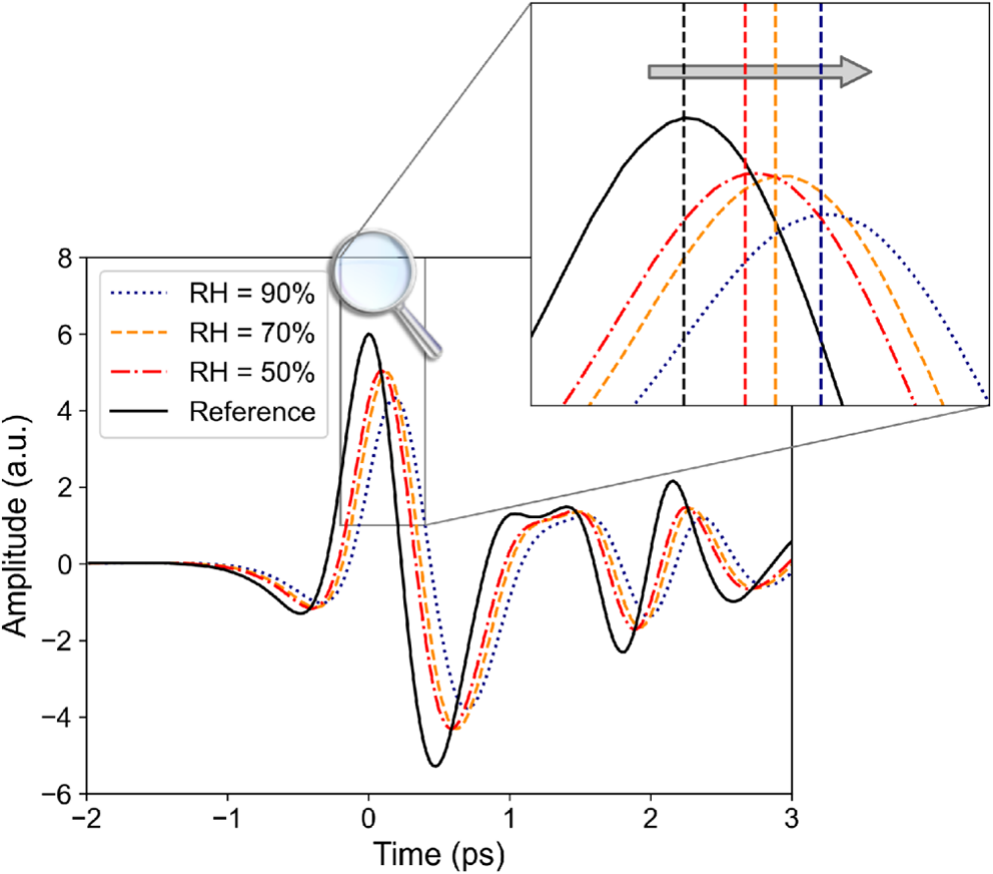
Time-resolved THz pulse amplitude for PEI/PAA films grown at 50%, 70%, and 90% RH, compared with a bare quartz substrate (black). The inset highlights the pulse maxima, showing systematic delays that depend on RH.

In the THz range, both the polymer matrix and absorbed water can contribute to the overall dielectric response of the films. Water is known to exhibit strong absorption in this frequency range due to its molecular dynamics,^50^ increasing the real part of the refractive index as hydration rises. The systematic dependence of the measured signal on relative humidity indicates that variations in water content play a dominant role in the observed changes. In addition to the temporal delay, the analysis also considers the attenuation of the THz signal, providing complementary information on absorption processes within the films. Furthermore, as the film thickness is comparable across all samples (Table 1), geometrical effects are unlikely to account for the observed variations.

**Table 1:** Morphological Properties and Self-Healing Behavior of PEI/PAA Films Grown at Different RH

RH	Thickness (μm)	Rq (μm)	Healing	Contact angle
50%	22 ± 4	1.8 ± 0.2	Visible scar remains	68°
70%	22 ± 3	2.1 ± 0.4	Visible scar remains	70°
90%	21 ± 3	14.8 ± 3.1	No residual damage	90°

The monotonic increase in delay from 50% to 90% RH directly supports the electrical results, confirming that multilayers deposited under higher humidity retain substantially more water. The THz-TDS measurements, therefore, reinforce the link between dielectric response, charge transport, and hydration established throughout this study.

### 3.4 Surface Morphology and Self-Healing

Self-healing behavior was evaluated qualitatively by introducing scratches and monitoring recovery after a 10 min immersion in water. Representative images (Figure 5a–f) show partial healing for films grown at 50% and 70% RH, which retained visible scars, whereas the 90% RH film exhibited complete healing with no residual damage. This enhanced healing is consistent with the higher water content, enabling greater polymer-chain mobility.

**Figure 5:**
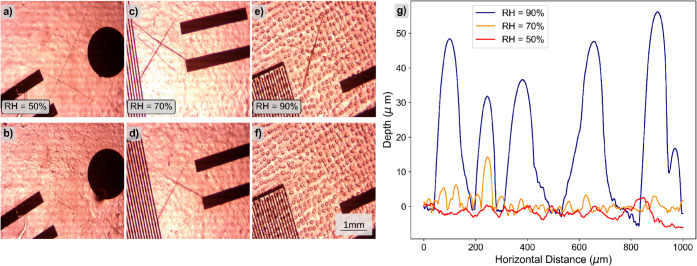
Self-healing and surface characterization of PEI/PAA films grown at different RH. (a–c) Scratched films before water immersion. (d–f) Same films after 10 min immersion. (g) Profilometry curves showing increased roughness at high RH.

Surface morphology was also strongly dependent on RH. Films grown at 90% RH exhibited circular surface domains and pronounced irregularities absent in the 50% and 70% samples. Profilometry (Figure 5g) quantified this trend: the root-mean-square roughness increased from 1.8 ± 0.2 μm (50% RH) to 2.1 ± 0.4 μm (70% RH) and 14.8 ± 3.1 μm (90% RH). Film thickness remained approximately constant at 21–22 μm across all samples. Interestingly, increased roughness correlated with higher water contact angles (Table 1), revealing that high-RH films not only self-heal more effectively, but also develop enhanced hydrophobicity, a functionality that emerges directly from humidity-controlled deposition. This behavior can be interpreted in the context of classical wetting models, where surface roughness either amplifies intrinsic wettability (Wenzel model) or promotes partial air entrapment at the interface (Cassie–Baxter model).^51,52^ Since the films are not intrinsically highly hydrophobic, the increase in contact angle observed at higher roughness suggests that surface structuring induces a transition toward a mixed wetting regime, in which partial air entrapment (Cassie–Baxter-like behavior) may contribute to the apparent hydrophobicity.

Such tunable wettability, controlled via deposition humidity, could be leveraged to optimize these films for applications in coatings, sensors, or flexible electronics, where surface interactions and self-healing performance are critical. It should be noted that the present evaluation focuses on morphological recovery. While the disappearance of visible damage indicates structural healing, the recovery of electrical functionality was not assessed in this work and remains an important topic for future investigation.

## 4 Conclusion

This study demonstrates that relative humidity during deposition plays a decisive role in defining the structural and functional properties of PEI/PAA multilayers, including electrical response, morphology, and self-healing behavior. Increasing RH from 50% to 90% leads to capacitance values nearly 5× larger, a stronger NDR peak centered at around 1.3 V, and THz-pulse delays rising from ≈91 fs to ≈183 fs, all consistent with higher water incorporation. Despite their similar thicknesses (21–22 μm), the films show pronounced structural changes: surface roughness increases from approximately 2 to 15 μm, and the water contact angle rises from 68° to 90°, revealing humidity-driven hydrophobicity. Self-healing performance is also strongly enhanced, with only the 90% RH film achieving recovery without scars after 10 min in water. These quantitative correlations establish RH as a powerful and accessible parameter for tailoring weak polyelectrolyte multilayers. The observed coupling between hydration and functional properties is relevant for the design of humidity-responsive coatings and soft electronic materials, although further studies are needed to evaluate performance in specific device configurations.

## #

Institute Lumière Matière—UCBL 1, Villeurbanne 69100, France
